# Essential role of sugar transporters BbStp13 in fungal virulence, conidiation, and cell wall integrity in entomopathogenic fungus *Beauveria bassiana*

**DOI:** 10.1080/21505594.2025.2563006

**Published:** 2025-09-18

**Authors:** Jinli Ding, Huiru Ling, Bin Li, Min Lu, Mingguang Feng, Shenghua Ying, Qing Cai

**Affiliations:** a Key Laboratory of Biocatalysis and Enzyme Engineering, School of Life Sciences, Hubei University, Wuhan, China; b Institute of Microbiology, College of Life Sciences, Zhejiang University, Hangzhou, China; c College of Plant Science and Technology, Huazhong Agricultural University, Wuhan, China

**Keywords:** Sugar transporters, conidiation, virulence, cell wall integrity, entomopathogenic fungi

## Abstract

Fungi primarily utilize sugar as energy sources, with their uptake facilitated by sugar transporters. *Beauveria bassiana* is an entomopathogenic fungus widely applied in pest biological control. This study identified 43 sugar transporter proteins in *B. bassiana*, of which 27 exhibited higher expression in aerial mycelia, indicating a greater reliance on sugar transport during aerial development. Notably, one transporter, BbStp13, demonstrated significantly elevated expression in aerial mycelia. Using homologous replacement and ectopic insertion techniques, BbStp13 deletion and complementation strains were constructed. The deletion of BbStp13 resulted in reduced virulence of *B. bassiana*, particularly during cuticle infection. Sugar transporters are also crucial for conidia development, a process essential for fungal dispersal. Deletion of BbStp13 impaired conidia production, especially under sugar-limited conditions. Furthermore, BbStp13 proteins mediate host–fungus interactions by modifying carbohydrate profiles on the fungal cell surface, thus influencing host immune recognition. Additionally, the BbStp13 protein is vital for maintaining cell wall homeostasis, contributing to the fungus’s resistance to host hemocoel stresses. In conclusion, the sugar transporter BbStp13 is central to maintaining fungal virulence, conidia development, cell wall integrity, and immune evasion during infection. This study broadens our understanding of sugar transporters in fungal physiology and provides deeper insights into the mechanisms underlying the lifestyle of entomopathogenic fungi. Furthermore, these findings identify potential targets for optimizing the efficacy of *B. bassiana* as a biological control agent, paving the way for more effective pest management strategies.

## Introduction

Carbohydrates (e.g. sugar) represent the most important organic substances absorbed by living organisms in quantitative terms [1]. They function as vital carbon frameworks for synthesizing diverse cellular components, serve as a key energy supply, and are instrumental in sustaining osmotic equilibrium within cells [2]. The processes of sugar uptake, exchange, and competition at biotrophic interfaces are managed by membrane transport proteins, which was named sugar transport [3]. In structural terms, about 99% of the sugar transporters belong to the major facilitator superfamily (MFS) and are included in the Transporter Classification Database, which contain 12 transmembrane domains [4]. In *Saccharomyces cerevisiae*, sugar transport Hxt proteins play a crucial role in the transport of various sugars and have been extensively studied for their dual function as transporters and partial sensors [5,6]. The diversity of sugar transporters and sensors, however, varies significantly across fungal species [7–10]. For example, genome sequencing of budding yeasts has uncovered 139 potential sugar transporters [9]. In filamentous fungi, the number of sugar transporters differs by species, with *Aspergillus niger* possessing 90 sugar transporters, *Aspergillus nidulans* 83, *Penicillium subrubescens* 117, and *Trichoderma reesei* 52 [10]. These transporters have evolved through processes of gain, loss, and diversification, reflecting each species’ adaptation to specific ecological niches and specialization in the uptake of particular sugar [11]. Fungal sugar transporters also display a high degree of functional redundancy and complexity [12]. Fungal genomes typically encode multiple sugar transporters with overlapping substrate preferences, ensuring that the organism can efficiently utilize available sugars in its environment [13,14]. Additionally, some transporters have broad specificity, capable of transporting a range of sugars [15]. For instance, in *Penicillium oxalicum*, three sugar transporters – CdtC, CdtD, and CdtG – facilitate the uptake of cellodextrin, while in *Aspergillus niger*, the well-studied sugar transporter MstA is known to transport several different sugars, including D-glucose, D-fructose, D-xylose, and D-mannose [13,15]. Despite these advances in understanding sugar transport in various fungi, research on sugar transporters in entomopathogenic fungi remains limited.

The entomopathogenic organism *B. bassiana* is crucial for managing arthropod populations and is extensively employed globally as a mycopesticide for pest control [16,17]. The infection process initiates when fungal spores, referred to as conidia, adhere to the cuticle of the insect host [18]. After attachment, the conidia germinate, enabling the fungus to produce invasive hyphae that penetrate the exoskeleton of the insect [19]. Once the exoskeleton is breached, the fungus accesses the hemocoel, which is an internal cavity abundant in carbohydrates sources [20]. At this point, *B. bassiana* undergoes a change, transforming into yeast-like hyphal structures (*in vivo* blastospores), which rapidly proliferate by drawing on the insect’s internal nutrients [21]. This depletion of nutrients ultimately causes the insect to perish [22]. After the host’s death, the fungus continues to proliferate on the carcass, generating a substantial amount of new conidia that can disperse and trigger further infection cycles [23]. A significant aspect of this infection process is the organism’s capacity to swiftly absorb and utilize carbon from the insect host. Like many filamentous fungi, *B. bassiana* prioritizes the consumption of preferred carbon sources – typically sugars – over other available nutrients [24]. The efficient uptake and metabolism of these sugars are critical to the fungus’s infectivity and virulence.

In this research, we discovered 43 sugar transport proteins in *B. bassiana*, which we classified into five unique groups. The expression profiles of these transport proteins demonstrated significant variation under aerial and submerged conditions. One notably high-expressed transporter was found in the aerial but not submerged conditions. To delve deeper into its function, we created gene deletion and complementation strains. Findings revealed that the sugar transporter, designated as BbStp13, was consistently localized within the cell membrane. The absence of BbStp13 adversely affected the development of *B. bassiana*, compromised the integrity of the cell wall, and diminished its virulence. This study underscores the critical role of sugar transport proteins in both the growth and pathogenicity of entomopathogenic fungi while also providing a foundation for future research into the functional roles these proteins play in pathogenic fungi.

## Methods and materials

### Strains and cultivation media

*B. bassiana* ARSEF2860 (Bb2860), a wild-type strain obtained from the US Department of Agriculture Entomopathogenic Fungus Collection (Ithaca, NY, USA), was used in this study. The fungal strains were grown on Sabouraud dextrose agar with yeast extract (SDAY), which contains 4% glucose, 1% peptone, 1.5% agar, and 1% yeast extract, at a temperature of 25°C. *Escherichia coli DH5α* (Invitrogen, Waltham, MA, USA) was cultured in Luria-Bertani (LB) medium with selective antibiotics, facilitating plasmid construction. Additionally, *Agrobacterium tumefaciens* strain *AGL-1*, which was used for fungal transformation, was grown in yeast extract broth (YEB) containing 0.5% sucrose, 1% peptone, 0.1% yeast extract, and 0.05% MgSO4. For fungal transformation and phenotypic assays, Czapek-Dox agar plates (CZA), consisting of 3% glucose, 0.3% NaNO_3_, 0.1% K_2_HPO_4_, 0.05% KCl, 0.05% MgSO_4_, 0.001% FeSO_4_, and 1.5% agar, were utilized.

### Bioinformatic analyses of sugar transport proteins in B. bassiana

The domain organization of sugar transporters was analyzed using the SMART online portal (https://smart.embl-heidelberg.de) [25]. Phylogenetic relationships between all identified proteins were established using MEGA software version 7, allowing for a deeper understanding of their evolutionary connections. To further explore the structural configurations of BbStp13 transporters, their three-dimensional structures were predicted using the AlphaFold online platform (https://colab.research.google.com/github/deepmind/alphafold/blob/main/notebooks/AlphaFold.ipynb).

### Sub-cellular localization of BbStp13 proteins in B. bassiana

The sub-cellular localization of BbStp13 was demonstrated following previously established methods [26]. All primers used in the study are listed in Supplementary Table S1. The coding sequence for *BbSTP13* was amplified using the primer pair BbStp13GF/BbStp13GR, and the resulting amplified fragment was cloned into the pBMGS vector, which had been digested with *BamH*I and *Xma*I. This process fused the *BbStp13* sequence to the 3′-end of the green fluorescent protein (GFP) gene. The resulting construct was then transformed into the wild-type strain of *Beauveria bassiana*, and the transformants were selected on Czapek-Dox agar (CZA) plates containing 15 μg/ml chlorimuron ethyl. To visualize the localization, the transformants were cultured on SDAY plate and the resultant mycelia/conidia were stained with calcofluor white (CFW) a fluorescent stain for fungal cell walls and CMAC (Vacuolar specific dye). The fluorescent signals were then observed using a confocal laser scanning microscope.

### Real-time PCR analysis

Real-time PCR analysis was employed to assess the expression pattern of the *BbSTP13* gene [27]. Conidial suspensions of the wild-type strain were inoculated onto SDAY plates and into SDB (which are SDAY plates without agar). Aerial mycelia were sampled for the first time at 1 d post-incubation, with subsequent samples taken daily up to 7 d. Submerged mycelia were sampled daily for a total of 3 d. Total RNA was extracted from the samples using TRIzol® Reagent (Sigma-Aldrich, Missouri, USA) in accordance with the manufacturer’s instructions. Complementary DNA (cDNA) was synthesized from the total RNA using the PrimeScript™ RT Reagent Kit (TaKaRa, Dalian, China). The resulting diluted cDNA was then used as a template for real-time PCR analysis, with the primer pair RTBbStp13F/RTBbStp13R targeting the *BbStp13* gene. As an internal standard, the 18S rRNA gene was amplified using the primer pair 18SF/18SR. All primers used in the experiments are listed in Table S1. The relative expression levels of the *BbStp13* gene were calculated using the 2^−ΔΔCT^ method, allowing for a comparison of gene expression across different conditions and time points [28]. Each assay was replicated four times to ensure accuracy and consistency of the results.

### Gene disruption and complementation

Gene disruption and complementation experiments were conducted utilizing homologous replacement and ectopic insertion techniques, respectively [29]. Details of all relevant primers can be found in Supporting Information Table S1. To amplify the flanking regions both upstream and downstream of the targeted gene, the primer pairs BbStp13UF/BbStp13UR and BbStp13DF/BbStp13DR were employed. The resultant amplified fragments were subsequently cloned into the p0380-bar vector at the *Xma*I/*BamH*I and *Xba*I/*Hpa*I restriction sites, making use of the ClonExpress II One-Step Cloning Kit (Vazyme Biotech, Nanjing, China). The gene disruption vectors that resulted from this process were labeled as p0380-bar-BbStp13. For creating complementation strains, the complete coding sequence of BbStp13 was amplified with the primer pair BbStp13HF/BbStp13HR and introduced into the p0380-sur vector. The complementation vectors produced were referred to as p0380-sur-BbStp13. Transformation of the fungal cells was performed through the Agrobacterium tumefaciens-mediated transformation method. To isolate potential disruption and complementation strains, Czapek-Dox agar (CZA) plates enriched with 200 μg/ml phosphinothricin and 15 μg/ml chlorimuron ethyl were utilized. Verification of candidate transformants was achieved via PCR analysis with the primer pair BbStp13JF/BbStp13JR, confirming the successful disruption or complementation of the targeted gene.

### Effects of the gene loss on fungal phenotypes

The 7 d conidia were collected and utilized as the primary inoculum for phenotypic evaluations. The phenotypic assessments were performed among the wild-type strain, the Δ*Bbstp13* mutant strain, and the complemented mutant strain, with each set of experiments carried out in triplicate [30].

#### Vegetative growth

A 1 µL sample of conidial suspension (10^6^ conidia/mL) was inoculated on modified Czapek-Dox agar (CzA) plates, with the carbon and nitrogen components adjusted according to experimental needs. The tested carbon sources (all at a final concentration of 3% w/v unless specified) included glucose, sucrose, trehalose, lactose, maltose, fructose, glycerol, and mannitol, in addition to olive oil (0.5%) and oleic acid (0.2%). The nitrogen sources evaluated included NH_4_Cl (35 mM), urea (0.5% w/v), chitin (0.5% w/v), gelatin (0.5% w/v), peptone (0.5% w/v), and NH_4_NO_3_ (0.5% w/v). Following a 7-day incubation period at 25°C, the colony diameters were recorded to determine the impact of different nutrient sources on fungal development.

#### Stress responses

Fungal tolerance to various stress conditions was assessed by growing the strains on CzA plates supplemented with stress-inducing agents. These included 2 mM H_2_O_2_ and 2 mM menadione (to induce oxidative stress), 0.5 M NaCl and 1 M sorbitol (to induce osmotic stress), as well as Congo red (3 μg/mL) and calcofluor white (CFW; 1 μg/mL) (to assess cell wall integrity stress). A 1 µL droplet of conidial suspension (10^6^ conidia/mL) was applied to the plates and incubated at 25°C. After 7 d of growth, the diameters of the colonies were measured. CzA plates without stress-inducing chemicals served as controls.

#### Aerial development

For the assessment of aerial development, a conidial suspension of 100 µL (10^7^ conidia/mL) of wild type, Δ*Bbstp13* deletion and complementation strains were inoculated onto modified SDAY plates. These plates were then incubated at a temperature of 25°C while varying the glucose concentrations at 1%, 2%, 3%, and 4%. After a period of 7 d, mycelial discs were collected into 0.02% tween 80, and after ultrasonic dispersion, and the quantification of conidial production was measured in terms of spores per cm^2^. For structural evaluation, conidiophores were stained using CFW and analyzed with a laser scanning confocal microscope (LSCM).

#### Blastospores yield

Blastospores were cultivated in modified SDB medium containing glucose concentrations of 1%, 2%, 3%, and 4%. Conidial suspensions were introduced at a final density of 10^6^ conidia/mL and grown at 25°C with continuous shaking for 2.5 d. The concentration of blastospores was assessed by counting the spores per mL in the broth.

#### Germination test

To assess conidial germination, water agar (WA) plates (1.5% agarose) served as the nutrient-limited medium, while sucrose-peptone agar (SPA) plates (2% sucrose, 0.5% peptone, 1.5% agar) constituted the nutrient-rich medium. Conidial suspension (500 µL, 5 × 10^7^ conidia/mL) was inoculated onto plates, and germination percentage was determined following incubation at 25°C.

#### Cuticle penetration

A 1 μL aliquot of conidial suspension (1 × 10^6^ conidia/mL) was applied to the center of an intact locust hindwing placed on CzA plate. After incubation at 26°C for 48 h, the wing was removed and the CzA plate was further incubated to permit fungal growth originating from hyphae that penetrated the wing and accessed the nutrient-rich medium. Successful penetration of the intact insect cuticle was indicated by colony emergence on the CzA plate within two days post-wing removal.

#### Conidial adherence

Conidial adherence assays utilized hind wings of Asian migratory locust (*Locusta migratoria manilensis*) surface-sterilized with 37% H_2_O_2_ for 5 min. To prevent potential surfactant-induced alterations to conidial surface properties, conidia harvested from each fungal strain were suspended in sterile water via vigorous vortex mixing. Each suspension was transferred into a Petri dish. Prepared hind wings were gently floated on suspension surfaces for 30 s to allow conidial attachment to the contacting side, followed by placement of the opposite side onto 0.7% water agar. After a 16-h incubation at 25°C, conidia within three randomly selected microscopic fields (50 µm^2^ each) per wing were quantified before and after removing less adherent conidia via 30 s sterile water washing. Each strain–substrate combination assay included three independent replicates.

### Conidial binding capacity to lectin

The characteristics of conidial lectin-binding were evaluated in accordance with established protocols [31]. To identify specific carbohydrate residues on the conidial surface, labeled lectins, such as Galanthus nivalis lectin (GNL) and wheat germ agglutinin (WGA) (from Vector Laboratories Inc., California, USA), were utilized. For the lectin-binding assessment, the conidia were initially suspended in a 0.02% Tween 80 solution and then fixed using a 3% (v/v) formaldehyde solution for a duration of 30 min. Following the fixation process, the conidia were re-suspended in a buffer and incubated in the dark with the fluorescently labeled lectins for one hour to ensure thorough binding. Subsequently, unbound lectins were washed away through multiple rinses to reduce background fluorescence.

### Phenol oxidase (PO) activity

PO activity was measured following the method. The substrate ortho-phenylenediamine (300 µL, 0.1 M) was diluted in 600 µL of PBS (0.06 M Na_2_HPO_4_, 0.04 M NaH_2_PO_4_, pH 7.0). A two-fold dilution of hemolymph (100 µL) was then added to the reaction mixture. The absorbance at 405 nm (OD_405_) was recorded every 30 s for a total of 6 min. PO activity was calculated as the change in OD_405_ per minute, with one unit of activity defined as a 0.001 change in OD_405_ per minute. Each assay was performed in triplicate.

### Fungal virulence assay using Galleria mellonella larvae

Fungal virulence was evaluated using *G. mellonella* larvae as bioassay hosts [32]. Each assay involved 30–35 insects, and each experiment was replicated three times to ensure statistical reliability. Two types of infection assays were conducted: the cuticle infection assay and the intra-hemocoel injection assay. In the cuticle infection assay, the larvae were immersed in a conidial suspension containing 10^7^ conidia/ml for 15 s, allowing the fungal spores to attach to the insect’s external surface. For the intra-hemocoel injection assay, a 5-μl conidial suspension (containing 10^5^ conidia/ml) was injected directly into the hemocoel of the host larvae. After infection, the insects were incubated at 25°C, and their mortality was monitored daily. The median lethal time (LT_50_), which represents the time required to kill 50% of the infected population, was calculated using the Kaplan – Meier method. A log-rank test was used to compare survival trends between different groups and assess statistical differences in virulence. Additionally, fungal development within the host hemocoel was assessed by quantifying the concentration of hyphal bodies in the hemolymph 3 d post-infection.

The hemocyte encapsulation assay was conducted following previously established methods. In brief, 5 μL aliquots of conidial suspension (containing 10^5^ conidia/ml) were injected directly into the hemocoel of the host organism. After injection, the infected hosts were maintained at 25°C to allow the fungal infection to progress. Hemolymph samples were collected from the hosts every 6 h after the 24 h. The interactions, specifically the process of hemocyte encapsulation (a host immune response), were observed and recorded using a microscope, allowing for detailed visualization of how the host immune cells responded to the fungal infection. Cadavers were maintained in humid Petri plates for 5 d, and fungal growth on cadavers was documented photographically. After 10 d of incubation, cadavers were transferred to 0.02% Tween 80. Following ultrasonication, conidia were suspended in Tween 80 and quantified microscopically.

### Statistical analyses

The comparison of the indicated phenotypes between the wild-type strain and the disruption mutant strain was analyzed using Student’s *t*-test. A *p*-value of less than 0.05 was considered statistically significant, indicating a meaningful difference between the paired phenotypes.

## Results

### Bioinformatic identification and expression modes of sugar transport domain – containing proteins

**Figure 1.**
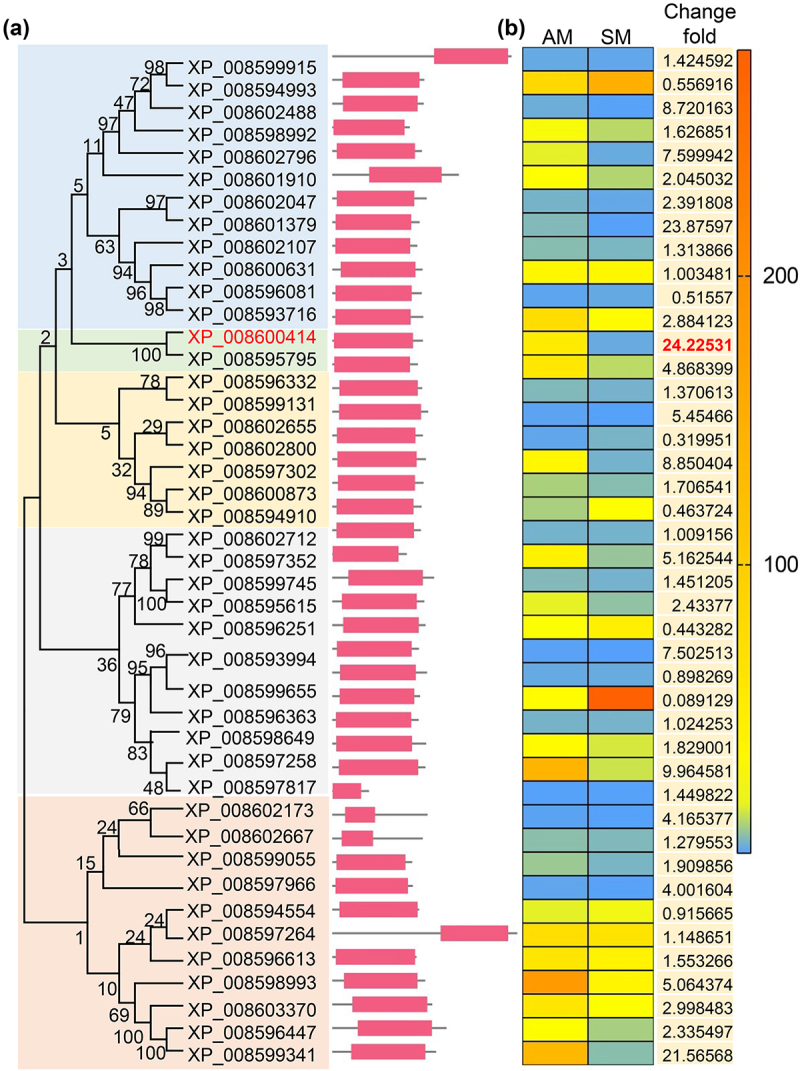
Bioinformatic identification of sugar transporters in *B. bassiana*.

Domain annotation revealed that *Beauveria bassiana* contains 43 proteins (BbStp1 through BbStp43) with sugar transporter domains, which are categorized into six distinct clusters (Figure 1(a)). Detailed structural information for these proteins is provided in Table S2. These 43 genes were expressed in both aerial (AM) and submerged (SM) mycelia, although their expression patterns showed significant differences between the two conditions (Figure 1(b)). Notably, *BbSTP13* (locus tag: BBA_07095), which encodes a protein with one sugar transporter domain and a low complexity region, was highly expressed in aerial mycelia. The expression level of *BbSTP13* in aerial mycelia was 24.23 times higher than in submerged mycelia, making it the most significantly altered sugar transporter protein under these conditions. Due to this substantial expression difference, *BbSTP13* was chosen for further investigation to determine its role in conidiation and blastospore formation.

(a) The relationships between *B. bassiana* sugar transporters were analyzed using the neighbor-joining method with 1,000 replicates. Each gene is labeled with its GenBank accession number, gene designation, and sugar transport domain architecture. Domain legends are displayed on the right side of the figure. (b) Transcriptional Levels of Sugar Transporter Proteins. The expression levels of sugar transporters were measured in aerial mycelia (AM) grown on SDAY and submerged mycelia (SM) grown on SDB medium. The heatmap color key is provided on the right side of the figure to indicate expression levels.

To explore the structural characteristics of sugar transporter domain-containing proteins, the structure of BbStp13 was predicted using the AlphaFold2 platform, known for its high accuracy in protein structure prediction. The model achieved a predicted Local Distance Difference Test (pLDDT) score of 83.3, indicating a reliable structural prediction (Figure. S1). The 3D model revealed that BbStp13 has a barrel-like structure formed by 12 α-helices, which aligns with the typical structural characteristics of sugar transporter proteins [33]. To explore the biological functions of BbStp13 proteins in *B. bassiana*, we created *BbSTP13* mutant strains using homologous recombination, and the complementation was accomplished through ectopic insertion (Figure. S2).

### Sub-cellular localization of BbStp13

**Figure 2.**
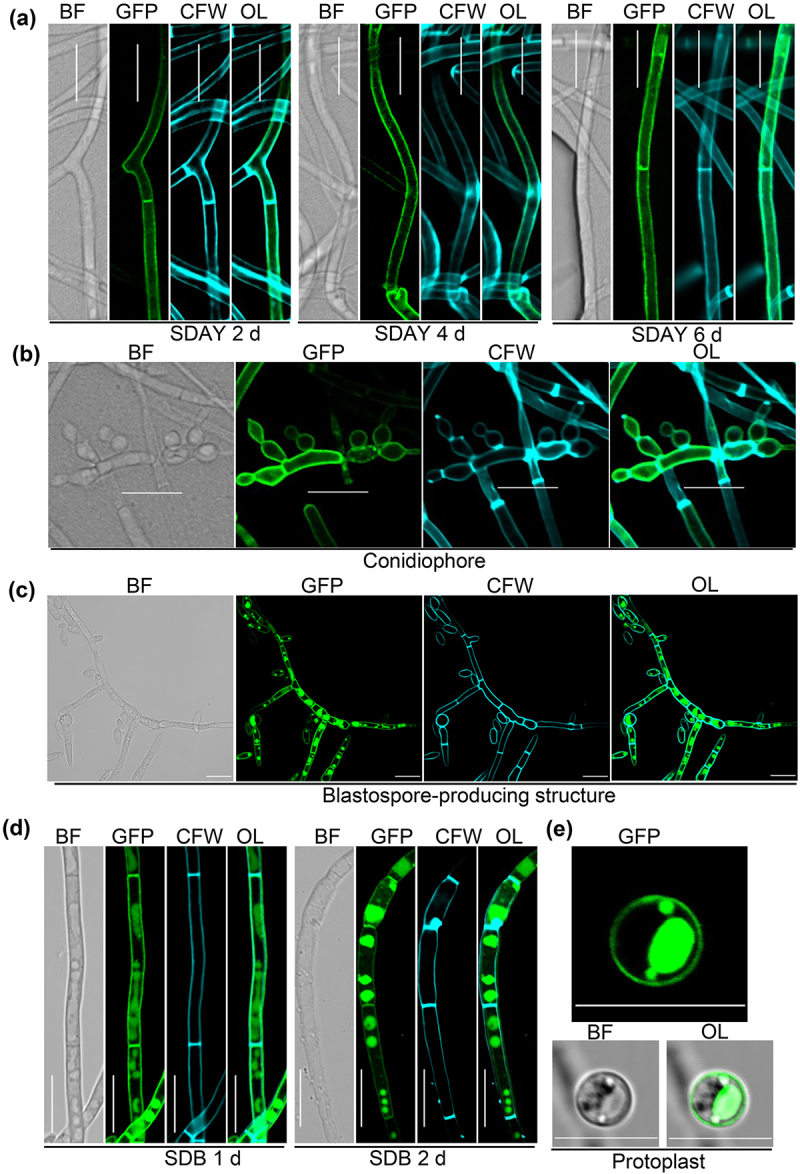
Sub-cellular imaging of BbStp13 in *B. bassiana*.

To determine the sub-cellular localization of BbStp13, a fusion gene (*BbSTP13-GFP*) was transformed into the wild-type strain of *B. bassiana*. The transformant was cultured on SDAY plates for 2, 4, and 6 d, and green fluorescent signals were consistently observed at the cellular periphery, coinciding with calcofluor white (CFW) staining (Figure 2(a)). In the conidiophore structure, green fluorescent signals were also seen at the cellular periphery (Figure 2(b)), and similar fluorescence was detected in structures involved in blastospore production (Figure 2(c)). When cultured in SDB medium for 1 and 2 d, the green fluorescent signals of BbStp13 overlapped with the blue fluorescence signal labeled with CWP (Figure 2(d)) and the red fluorescence signal labeled with CMAC (Figure. S3), indicating that Bbstp13 is localized cellular periphery and in vacuoles under submerged condition. In a protoplasting assay, GFP fluorescence was observed at the cell peripheries (Figure 2(e)). These findings suggest that BbStp13 is primarily associated with the cell membrane during fungal growth and development under both aerial and submerged conditions.

Sub-cellular localization of BbStp13 was investigated by fusing the protein coding sequence with a GFP gene, followed by transformation into the wild-type strain. The transgenic strain was cultured on SDAY plates and in SDB medium, and the resultant mycelia were stained with calcofluor white (CFW). (a) The transformant was cultured on SDAY plates at 25°C, and mycelia were sampled at the indicated time points. (b) Distribution of BbStp13 within conidiophore structures was examined. (c) Localization of BbStp13 in blastospore-producing structures was observed. (d) The transformant was cultured in SDB medium at 25°C. (e) Protoplast preparation experiment: 2-day-old mycelia were used to generate protoplasts using cell-wall degradation enzymes. Fluorescent signals were visualized using a laser scanning confocal microscope. BF:bright field; OL: overlapped. Scale bar: 10 µm.

### BbSpt13 proteins involve in B. bassiana development

Fungal development was evaluated on modified Czapek-Dox Agar (CzA) plates that included a range of carbon and nitrogen sources to analyze the growth characteristics of the wild-type strain (WT), the Δ*Bbstp13* mutant, and the complemented strains. As shown in Figure. S4(a) and (b), the deletion mutants showed no notable differences in colony diameter when compared to the wild-type strain.

**Figure 3.**
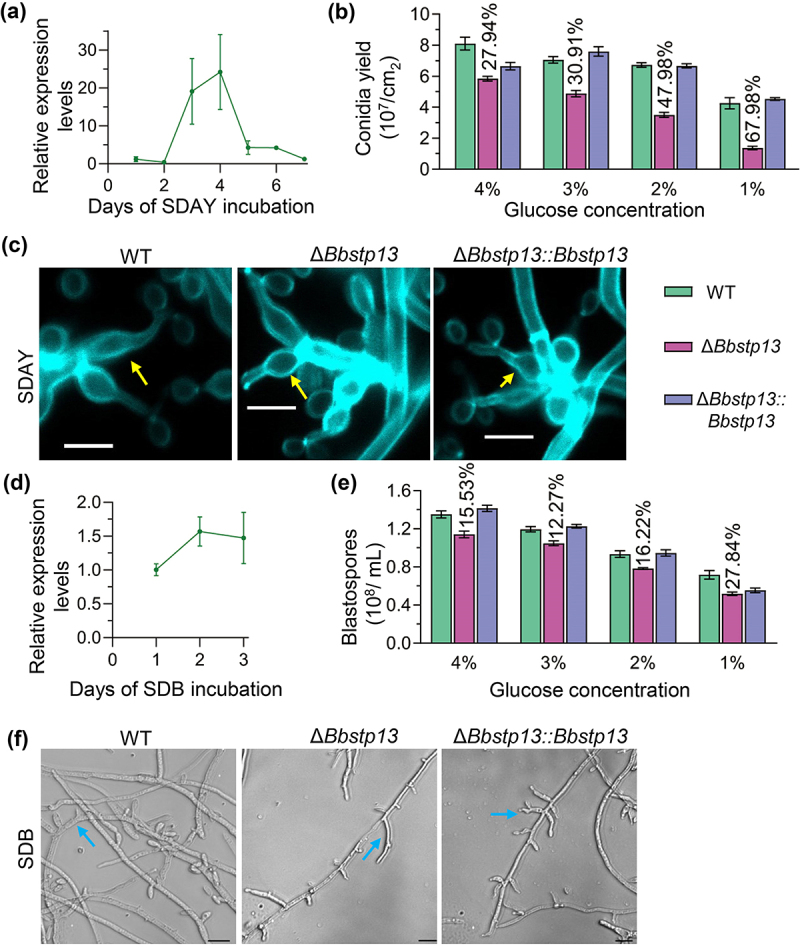
Impact of BbStp13 on development in *B. bassiana.*

On SDAY plates, *BbSTP13* displayed a fluctuating expression trend, peaking at 3 and 4 d post-incubation (Figure 3(a)). To assess the influence of gene disruption on conidiation, various concentrations of glucose were tested. There was no significant difference in the growth diameter Figure. S4(c) or biomass Figure. S4(d) between the Δ*Bbstp13* mutant, wild-type and complemented strains when cultured on a glucose medium with concentrations ranging from 1% to 4%. As shown in Figure 3(b), at a glucose level of 4%, the conidia yield of deletion strain was 5.84 ± 0.22 × 10^7^ conidia/cm^2^ (mean ± standard deviation), which was a 27.94% reduction compared to the wild-type strain (8.10 ± 0.58 × 10^7^ conidia/cm^2^). With a decline in glucose concentration, the gap in conidial production between the deletion strain and the wild-type became more apparent, with a 30.91% decrease at 3% glucose, 47.98% at 2%, and 67.98% at 1%. After four days of cultured on SDAY plates, the conidiophores of all strains were examined. The results showed no significant structural differences in conidiophores between the deletion strain and the wild-type strain (Figure 3(c)).

(a) RT-PCR analysis of *BbSTP13* expression during 7 d of incubation on SDAY plates. The relative expression levels were normalized to the expression at 1 d post-incubation. (b) Conidia production of the wild-type, *BbSTP13* deletion, and complementation strains was measured on SDAY medium with varying glucose concentrations (1% to 4%). (c) Conidiophores of the tested strains were sampled 4 d post-incubation on SDAY plates at 25°C, stained with calcofluor white (CFW), and visualized using a fluorescence microscope. Scale bar: 5 μm. (d) Expression level of BbStp13 in SDB medium was analyzed. (e) Blastospore production of the strains was measured in SDB medium containing different glucose concentrations (1% to 4%). (f) Morphological observation of blastospore-producing structures. Scale bar: 10 μm.

*BbSTP13* expression levels remained relatively stable across different culture time (Figure 3(d)). However, in media with diverse glucose concentrations, blastospore production by the deletion strain was consistently reduced compared to the wild-type strain. Specifically, in SDB media with glucose levels of 4%, 3%, 2%, and 1%, the deletion strain’s blastospore production decreased by 15.54%, 12.37%, 16.22%, and 27.84%, respectively (Figure 3(e)). Despite the reduced blastospore production, the structural of the blastospores produced by the deletion strain remained similar to those produced by the wild-type and complemented strains (Figure 3(f)). These findings demonstrate that while the disruption of BbStp13 did not significantly affect vegetative growth or conidiophore and blastospore-formation structure, it led to a marked reduction in both conidial and blastospore production, particularly under lower glucose concentrations. This suggests that BbStp13 plays a crucial role in regulating development in response to sugar availability.

### BbSpt13 proteins are involved in fungal virulence

**Figure 4.**
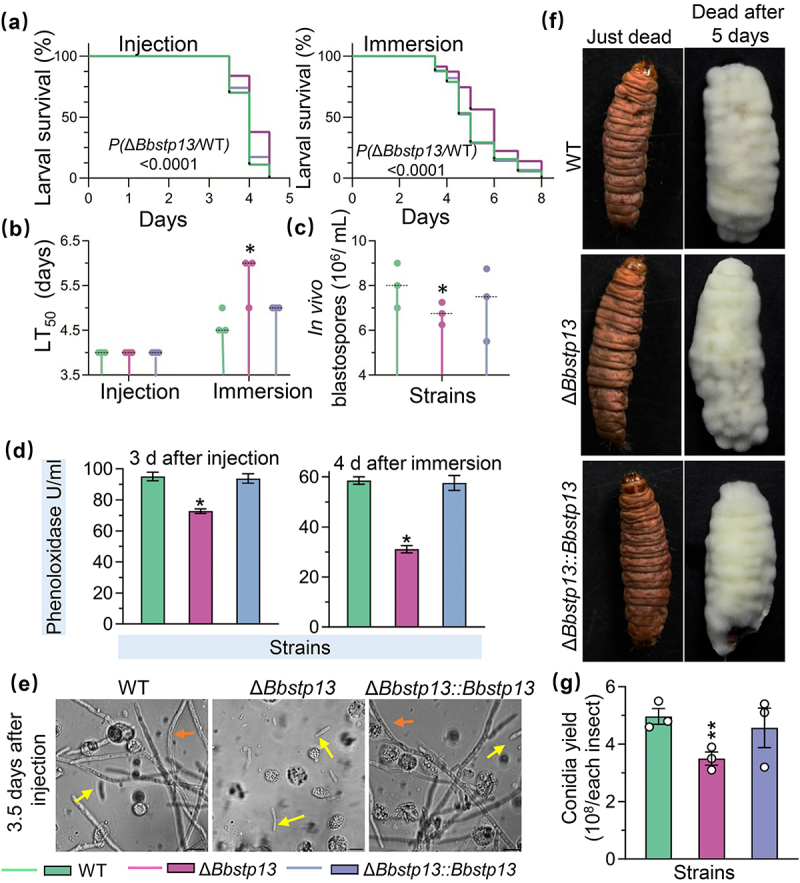
Effect of BbStp13 on fungal pathogenic growth and virulence.

Survival analysis indicated a decrease in host survival that is time-dependent across both inoculation methods Figure 4(a,b). For the wild-type strain, the median lethal time (LT_50_) was observed to be 4 d with the intra-hemocoel injection method and 4.67 d for the cuticle inoculation approach. In the bioassay using the intra-hemocoel injection, although the LT_50_ values for the Δ*Bbstp13* mutants was comparable to those of the wild-type strain, a notable decline in survival was evident in the survival curves of the Δ*Bbstp13* mutants when contrasted with the wild-type. Notably, in the cuticle inoculation approach, the LT_50_ for the Δ*Bbstp13* mutants was postponed by 1 d in comparison to the wild-type strain, suggesting a slower infection progression in the mutant strain. Additionally, the disruption of the *BbSTP13* gene led to a marked decrease in the formation of *in vivo* blastospore (Figure 4(c)). At 3 d following injection, the wild-type strain yielded 8.10 ± 0.58 × 10^6^ spores/mL, while the Δ*Bbstp13* mutants yielded 5.84 ± 0.22 × 10^6^ spores/mL, which reflects a 27.94% reduction compared to the wild-type strain. As shown in Figure 4(d), in virulence assay, the BbStp13 deletion strain caused a significant repression in phenoloxidase activity in the host hemolymph when compared with the wild-type strain. At 3.5 d post-injection, both the wild-type and complemented strains had produced a large number of yeast-like hyphal bodies and extended mycelium (Figure 4(e)). On the other hand, the Δ*Bbstp13* deletion strain was restricted to producing only yeast-like hyphal bodies, lacking the development of extended mycelium. After developing in humid conditions for 5 d, the mycelia on cadavers of Bbstp13 deletion strain compared with wild-type and complemented strains were no different (Figure 4(f)). After 10 d of cultivation, the BbStp13 deletion strain produced 3.50 ± 0.33 conidia/per insect on the infected larvae, a decrease of 29.53% compared to the wild strain (4.97 ± 0.39 conidia/per insect) (Figure 4(g)). These results underscore the importance of BbStp13 in ensuring the full virulence of *B. bassiana*.

(a) Fungal virulence was assessed using two methods: intrahemocoel injection (500 conidia per host) and topical inoculation (15-s immersion in a 10^7^ conidia/mL suspension). (b) The survival percentage of hosts was recorded daily, and the median lethal time (LT_50_) was calculated using Kaplan – Meier analysis. (c) *in vivo* blastospore yield was measured 3 d post-infection to evaluate fungal proliferation within the host. (d) Phenoloxidase (PO) activity was measured at 3 d post infection and 4 d after immersion. (e) Microscopic examination of *in vivo* blastospores was conducted at 3.5 d post-infection to observe fungal development within the host. (f) Images of fungi-killed larvae (fresh cadavers) and fungal outgrowths 5 d post-death. (g) The yield of conidia on the cadavers of each insect. Asterisks indicate significant differences in colony diameters between wild-type and BbStp13 disruption mutant strains (Student’s *t*-test, *P < 0.05, **P < 0.01).

### BbStp13 plays a crucial role in germination under poor nutrition and adherence to onion epidermis

**Figure 5.**
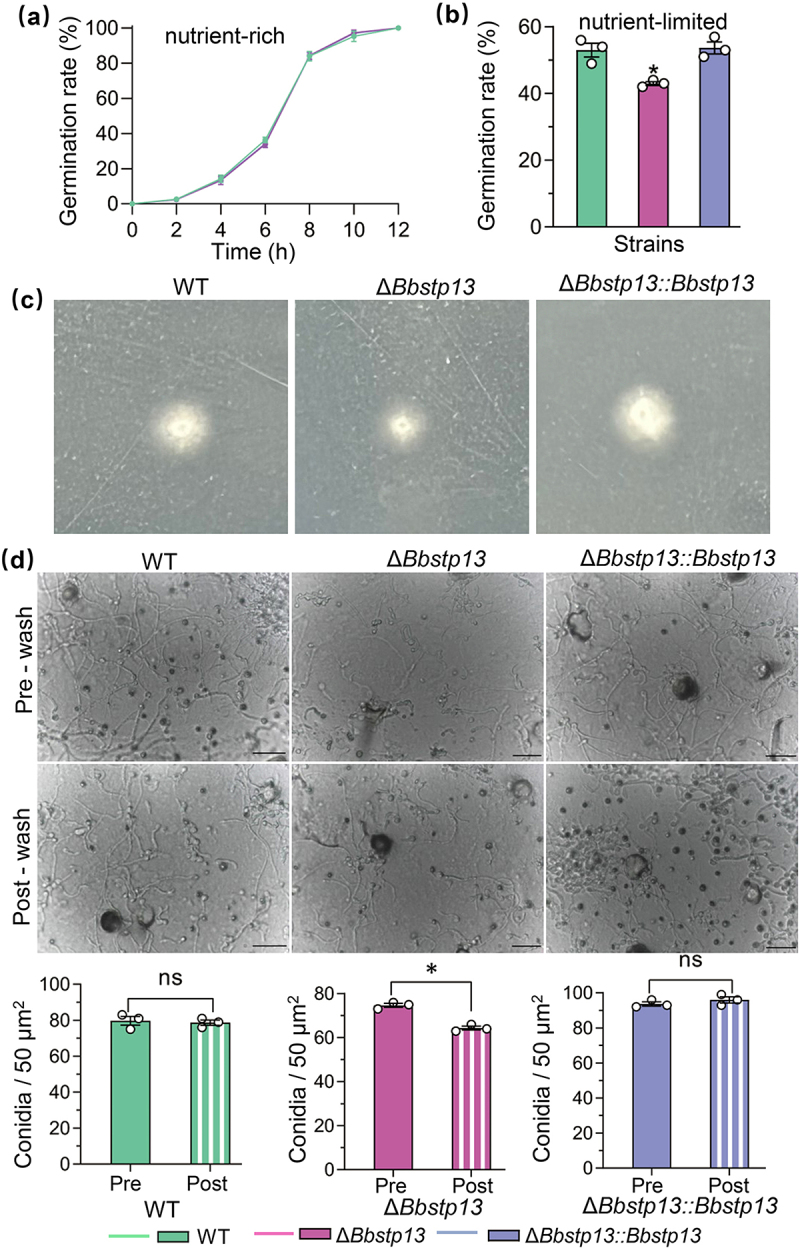
Experimental for conidial germination, cuticle penetration, and adherence assays.

Under normal nutritional conditions, no significant difference in germination rate was observed between the BbStp13 deletion strain and the wild-type strain (Figure 5(a)). However, under nutrient-poor conditions, the germination rate of the deletion strain on water agar after 24 h of culture was 43 ± 0.82%, representing an 18.88% decrease compared to the wild-type strain (53 ± 2.94%) (Figure 5(b)). The Δ*Bbstp13* strain can penetrate the insect cuticle, same with the wild-type and complementation strains successfully breached the cuticle (Figure 5(c)). BbStp13 influences conidial adhesion to locust hind wings. Following application of conidial suspensions onto locust wings and 16 h of cultivation, conidia within a 50 μm^2^ area were quantified pre-wash and post-wash. As shown in Figure 5(d), no significant difference in conidial retention was detected between the wild-type and complementation strains pre-wash and post-wash. In contrast, the number of adhered conidia in the Bbstp13 knockout strain was significantly reduced post-wash.

(a, b) Conidial germination assays: Aliquots (500 µL) of conidial suspension (5 × 10^7^ conidia/mL) were inoculated onto SPA nutrient medium plates and water agarose plates, respectively, and incubated at 25°C. Germination percentages are shown for each medium. (c) Cuticle penetration assay: Conidia were applied to locust hindwings placed on CzA plates and incubated at 26°C for 48 h to enable penetration. Hindwings were subsequently removed, and plates were incubated at 25°C to assess fungal growth. The emergence of a fungal colony indicates successful cuticle penetration. (d) Conidial adherence assay: Microscopic images of conidia attached to locust hindwings after 16 h incubation at 25°C, pre-wash and post-wash. Adherence was quantified as post-wash and pre-wash conidial counts. Asterisks denote significant differences between wild-type and BbStp13 disruption mutant strains (Student’s t-test, *P < 0.05). Scale bar: 10 μm.

### BbSpt13 responses to cell wall-perturbing stress

**Figure 6.**
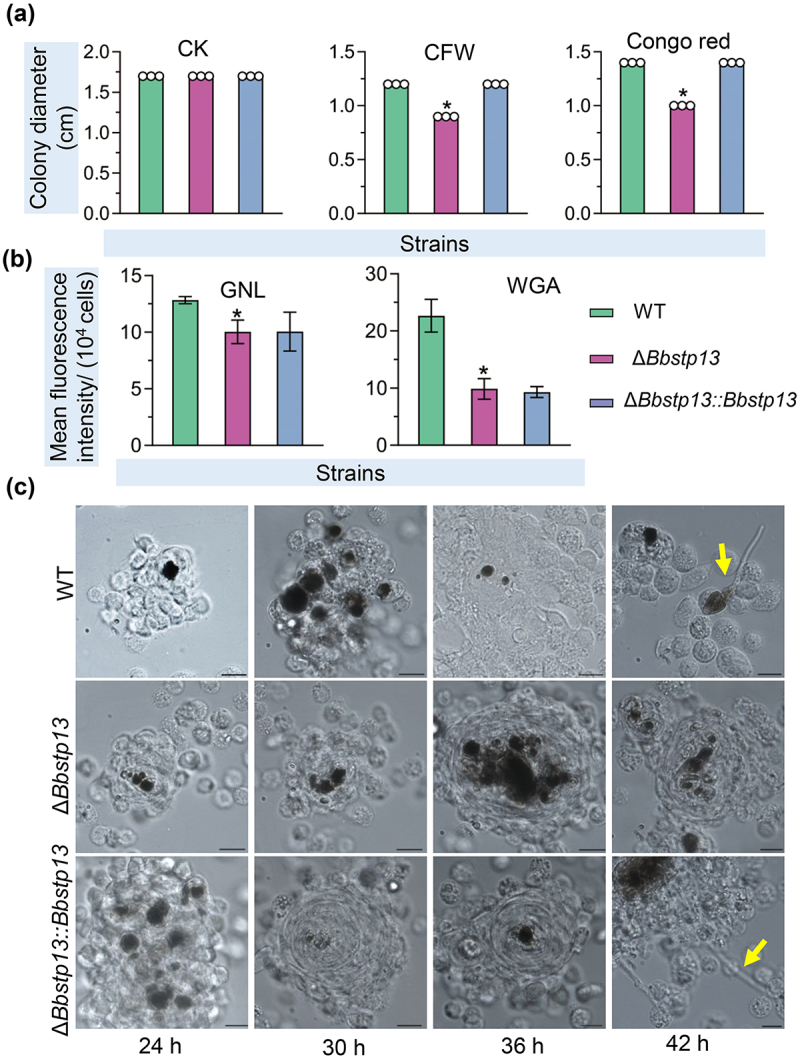
BbStp13 aids in fungal resistance to cell wall-perturbing agents and host recognition.

On the CzA plates, the colony diameters of Δ*Bbstp13* deletion mutants were consistent with those of the wild-type and complemented strain (Figure 6(a)). However, when 1 μg/mL CFW was added to the medium, the colony diameters of the mutant (0.9 cm) were significantly reduced compared to the WT strain (1.2 cm). Similarly, when the medium was supplemented with 3 μg/mL Congo red, the colony diameter of the Δ*Bbstp13* mutants measured 1.0 cm, reflecting a 28.57% reduction compared to the WT strain (1.4 cm). In contrast, when H_2_O_2_, menadione, NaCl, or sorbitol were added to the medium, the colony diameters of the Δ*Bbstp13* mutants did not show a significant difference from those of the WT strain (Figure. S4(e)). This suggests that the Δ*Bbstp13* mutants are particularly sensitive to cell wall stress.

(a) Resistance to cell wall – perturbing stresses was tested on CzA plates supplemented with Congo Red (CR) and Calcofluor White (CFW). A 1 µL conidial suspension was cultured at 25°C, and colony diameters were measured 7 d post-incubation. (b) Conidial lectin-binding traits were analyzed using flow cytometry. Lectins, including wheat germ agglutinin (WGA) and Galanthus nivalis lectin (GNL), were labeled with fluorescent dye to examine binding. (c) Conidial suspensions (5 µL, 10^5^ spores/mL) were injected into the host hemocoel, and hosts were incubated at 25°C. Hemolymph samples were taken at 24 h and then every 6 h. Hemocyte encapsulation was observed following fungal invasion. At 42 h post-infection, wild-type and complemented strains produced free-floating hyphal bodies, while BbStp13 deletion strains failed to escape from hemocyte encapsulation. Asterisks indicate significant differences in colony diameters between wild-type and BbStp13 disruption mutant strains (Student’s *t*-test, *P < 0.05).

### BbStp13 plays a crucial role in conidial lectin-binding activity and evasion from host cellular immunity

Flow cytometry analysis revealed that deletion of the *BbSTP13* gene significantly altered the lectin-binding properties of conidia. Specifically, the mutant strains exhibited a reduced ability to bind Galanthus nivalis lectin (GNL) and wheat germ agglutinin (WGA) (Figure 6(b)). In the wild-type strain, the mean fluorescence intensity for GAL and WGA was 12.83 ± 0.45 and 22.66 ± 4.05, respectively. In contrast, the fluorescence intensity in the BbStp13 deletion strain was 10.03 ± 1.48 for GAL and 9.88 ± 2.55 for WGA, representing decreases of 21.82% and 56.39%, respectively, compared to the wild-type strain.

In response to fungal invasion, hemocytes in the host exhibited clustering behavior and the formation of melanic dots. As shown in Figure 6(c), no noteworthy differences in hemocyte responses were observed between the wild-type strain and the disruption mutants at 24, 30, and 36 h post-infection (HPI). Nevertheless, by 42 HPI, the wild-type and complemented strains managed to escape from the clusters of hemocytes and initiated the production of hyphal bodies. Conversely, the disruption mutants of the *BbSTP13* gene failed to avoid the host hemocytes during this period, suggesting a delay in both immune evasion and fungal progression.

## Discussion

Fungi preferentially utilize mono- and disaccharides over other carbon sources as their primary energy substrates [24]. Since sugars cannot freely cross biological membranes, their uptake into fungal cells requires specialized sugar transporters [34]. This study focuses on identifying 43 proteins with sugar transporter domains in entomopathogenic fungi and analyzing their expression patterns under both aerial and submerged mycelia. Interestingly, the expression levels of the majority of these transporters (27 proteins) were found to be higher in aerial mycelia compared to submerged mycelia, indicating that fungi may rely more heavily on sugar transporters during aerial development. To explore this further, we conducted a detailed analysis of *BbSTP13*, a sugar transporter that exhibited significantly elevated expression in aerial mycelia compared to submerged mycelia.

The diversity of sugar transporters found in fungi is significant, demonstrating their ability to adjust to various environments [35,36]. For instance, *S. cerevisiae* cannot metabolize pentose sugars because it lacks the requisite transporters in its genome [34]. Nevertheless, this yeast has developed an expanded array of hexose transporters (Hxt), which enables it to flourish in environments rich in glucose [37]. On the other hand, the filamentous fungus *A. niger* possesses a more intricate system, featuring 86 anticipated sugar transporters categorized into nine subfamilies. This variability in the expression and regulation of sugar transporters is likely crucial for *A. niger*‘s success in inhabiting diverse habitats and its notable nutritional flexibility [12]. About 29 and 85 sugar transporters were found in the genome of the fungus *Metarhizium robertsii* and *Cordyceps militaris* [38,39]. Likewise, *B. bassiana* has 43 sugar transporter proteins. The varying expression profiles of these proteins indicate that they fulfill physiological roles at distinct times, allowing *B. bassiana* to adjust to different environmental conditions throughout its life cycle, especially as it shifts between parasitic and saprotrophic growth stages. BbStp13, as a representative protein in the sugar transporter domain, is vital for the virulence of fungi, which is crucial for the biocontrol capabilities of entomopathogenic fungi. Throughout its infection cycle, *B. bassiana* faces different nutritional environments, and its ability to quickly adapt to available nutrients is crucial for maintaining infectivity [18,19]. Sugars, as easily metabolizable nutrients, are typically the first to be utilized by the fungus [24]. As a result, the absence of functional sugar transporters can negatively impact *B. bassiana*‘s infectivity. The BbStp13 deletion strain exhibited a slight reduction in virulence for both immersion and injection infections, with a more marked decrease observed in immersion infection virulence compared to that of injection infection. This is mainly due to the oligotrophic conditions present in the body wall, where a greater number of sugar transporters is necessary to absorb limited sugar molecules. The BbStp13 deletion strain exhibited a slight decrease in virulence and this limited effect on virulence is likely attributed to the functional compensation by other homologous sugar transport proteins, which may take over the role of BbStp13. This redundancy likely explains why BbStp13 deficient strains did not experience growth defects when cultured on different sugar media, as other transporters were able to maintain the fungal adaptation and nutrient uptake necessary for growth. In the plant-symbiotic fungus *M. robertsii*, MST1 (monosaccharide transporter) is not involved in infection virulence but important for rhizoplane colonization. This phenomenon is caused by the functions of MST1 are compensated for by other genes in the Δ*Mst1* deletion mutant during infection of insects [40].

The sugar metabolic pathway plays a vital role in the conidiation process of entomopathogenic fungi. In *Fusarium oxysporum f. sp. cucumerinum*, galactofuranose (Galf)-containing sugar chains are essential for both hyphal growth and conidiation [41]. Similarly, in *B. bassiana*, the enzymes Trehalose-6-phosphate synthase (TPS) TpsA and TpsB are critical for regulating conidiation capacity and conidial quality, which are crucial for the fungus’s survival and propagation [42]. In *A. fumigatus*, the catalytic enzymes glucokinase and hexokinase play key roles in sugar metabolism and are essential for germination, growth, and conidiation [43]. The deletion of the *BbSTP13* gene in *B. bassiana* has been shown to impair the fungus’s ability to produce conidia, especially under conditions where sugar utilization is limited. This restriction in sugar uptake directly impacts the fungus’s reproductive capabilities and its ability to colonize new environments. This limitation is particularly pronounced during the saprotrophic phase on host cadavers, where external sugar sources are scarce. Therefore, efficient sugar absorption becomes crucial for successful fungal dispersal, and the continuation of its life cycle in challenging nutrient environments.

During interactions with the host, fungi are capable of invading host cells and triggering immune defense responses [31]. In *B. bassiana*, the BbStp13 proteins play a crucial role in mediating these host–fungus interactions. Pathogenic fungi typically display various carbohydrates (e.g. β‐1,3‐glucan, α‐1,6‐glucan, and β‐1,6‐glucan) on their cell surfaces, which are involved in pathogen recognition and the initiation of immune reactions [44]. Similarly, *B. bassiana* exhibits stage-specific carbohydrate profiles on its cell surface [45]. Modifications in the carbohydrate composition of fungal cell walls can impact the host’s immune recognition [46]. In *M. oryzae*, deficiencies in mannose transport may lead to issues in the biosynthesis of cell wall mannoproteins, resulting in altered cell wall structure and diminished pathogenic ability [47]. In the BbStp13 deletion strain, the lectin-binding properties of conidia are significantly altered, indicating a change in surface carbohydrate composition. In *B. bassiana*, five BbLTA proteins function to mask these carbohydrate components in the cell wall, preventing immune recognition and subsequent activation of the host’s immune system [32]. The BbStp13 proteins also contribute to maintaining cell wall homeostasis, as evidenced by the strain’s reduced resistance to Congo Red and calcofluor white, both of which are essential for the development of host hemocytes. Moreover, enzymes such as Trehalose-6-phosphate synthase (TPS) TpsA and TpsB are crucial for maintaining cell wall integrity and virulence in *B. bassiana* [42]. Similarly, in *Magnaporthe oryza*e, two nucleotide sugar transporters are critical for cell wall stability and full virulence [47]. This functional link between sugar transport and cell wall integrity is vital for the pathogen’s ability to infect, persist, and cause disease in its host.

Overall, this study highlights the crucial role of sugar transporters in the physiology and pathogenicity of *B. bassiana*, with a focus on BbStp13. BbStp13 plays a key role in cell wall integrity, host interactions and fungal virulence. Its deletion significantly impairs conidial production, particularly under sugar-limited conditions. These findings offer valuable insights into the roles of sugar transporters in entomopathogenic fungi, deepening our understanding of the virulence mechanisms facilitated by these transporters in pathogenic fungi.

## Supplementary Material

Supplemental_data - Clean.docx

## Data Availability

All datasets generated for this study are included in this article and the supplementary files. The data that support the findings of this study are available in figshare at https://doi.org/10.6084/m9.figshare.27184650.
