# Supplementary material for: Essential role of sugar transporters BbStp13 in fungal virulence, conidiation, and cell wall integrity in entomopathogenic fungus Beauveria bassiana
Source: Virulence. 2025 Sep 18;16(1):2563006. doi: 10.1080/21505594.2025.2563006 (PMC12533951; doi:10.1080/21505594.2025.2563006)
Supplement: Supplemental_data - Clean.docx [file KVIR_A_2563006_SM2860.docx]

**Essential role of sugar transporters BbStp13 in fungal virulence, conidiation, and cell wall integrity in entomopathogenic fungus *Beauveria bassiana***

Jinli Ding^1, 2^*, Huiru Ling^1^, Binli^1^, Minlu^1^, Mingguang Feng^2^, Shenghua Ying^2^, Qing Cai^3^*.

1 Key Laboratory of Biocatalysis and Enzyme Engineering, School of Life Sciences, Hubei University, Wuhan, 430062, China.

2 Institute of Microbiology, College of Life Sciences, Zhejiang University, Hangzhou, 310058, China

3 College of Plant Science and Technology, Huazhong Agricultural University, Wuhan, 430070, China

*Corresponding author:

Jinli Ding, Key Laboratory of Biocatalysis and Enzyme Engineering, School of Life Sciences, Hubei University, Wuhan, 430062, China. Email: dingjinli1007@outlook.com

Qing Cai, College of Plant Science and Technology, Huazhong Agricultural University, Wuhan, 430070, China. Email: caiqing@mail.hzau.edu.cn


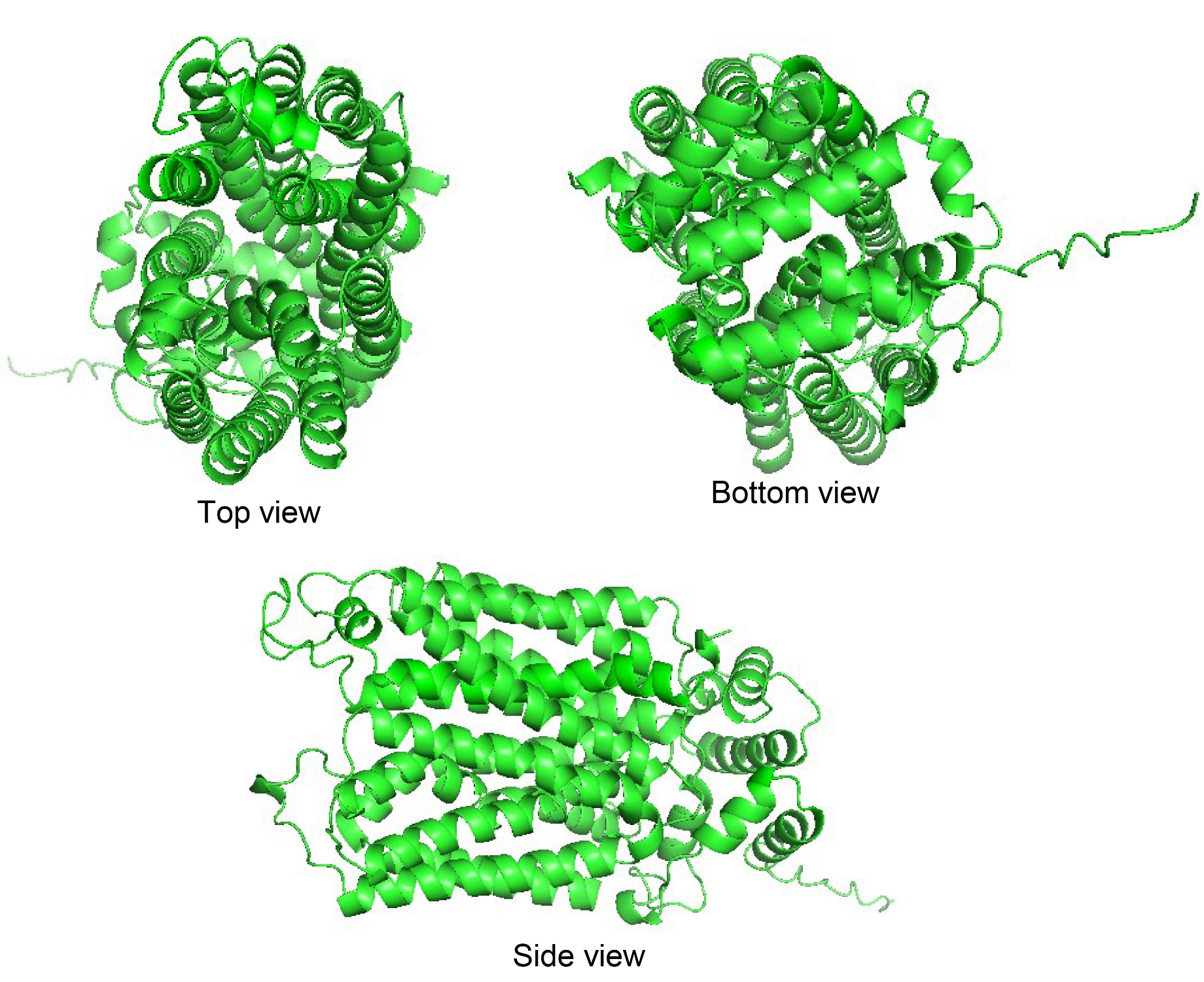


**Figure S1 Structural analysis.** The protein structure was predicted by online AlphaFold. The 3D model revealed that BbStp13 has a barrel-like structure formed by 12 α-helices.


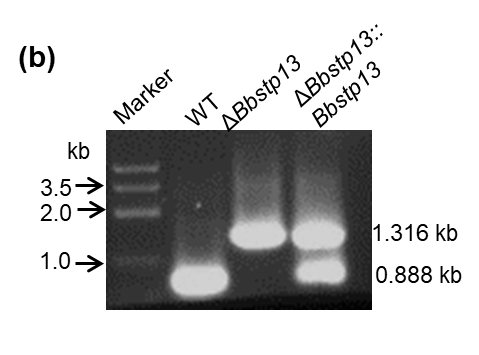

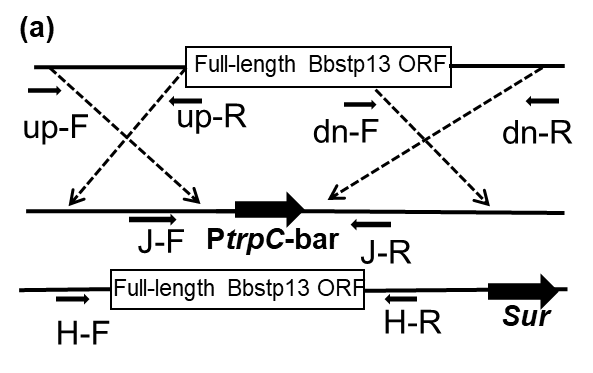


**Figure S2 Gene disruption and complementation in *B. bassiana*.** (a) A diagram deciphering primer design for gene disruption and complementation. Gene disruption and complementation were performed via target replacement and ectopic insertion, respectively. (b) PCR validation of recombination events.


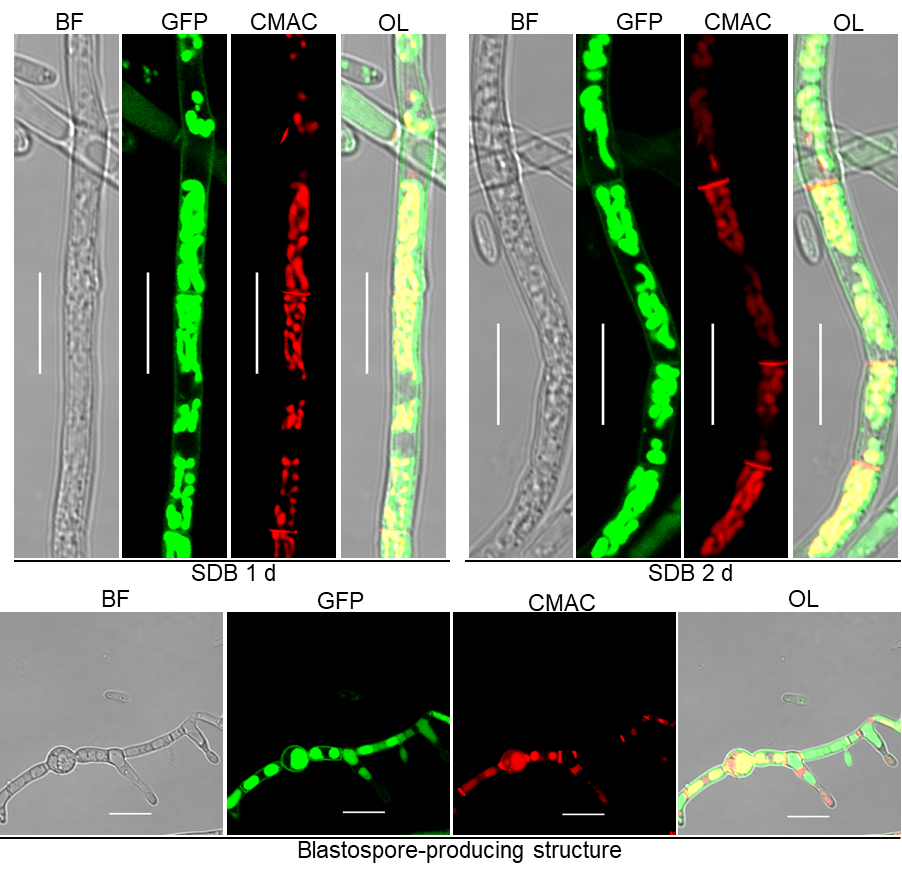


**Figure S3. Sub-cellular imaging of BbStp13 in *B. bassiana*.** Sub-cellular localization of BbStp13 was investigated by fusing the protein coding sequence with a GFP gene, followed by transformation into the wild-type strain. The transgenic strain was cultured in SDB medium, and the resultant mycelia were stained with CMAC (Vacuolar specific dye). Fluorescent signals were visualized using a laser scanning confocal microscope. BF:bright ﬁeld; OL: overlapped. Scale bar: 10 µm.


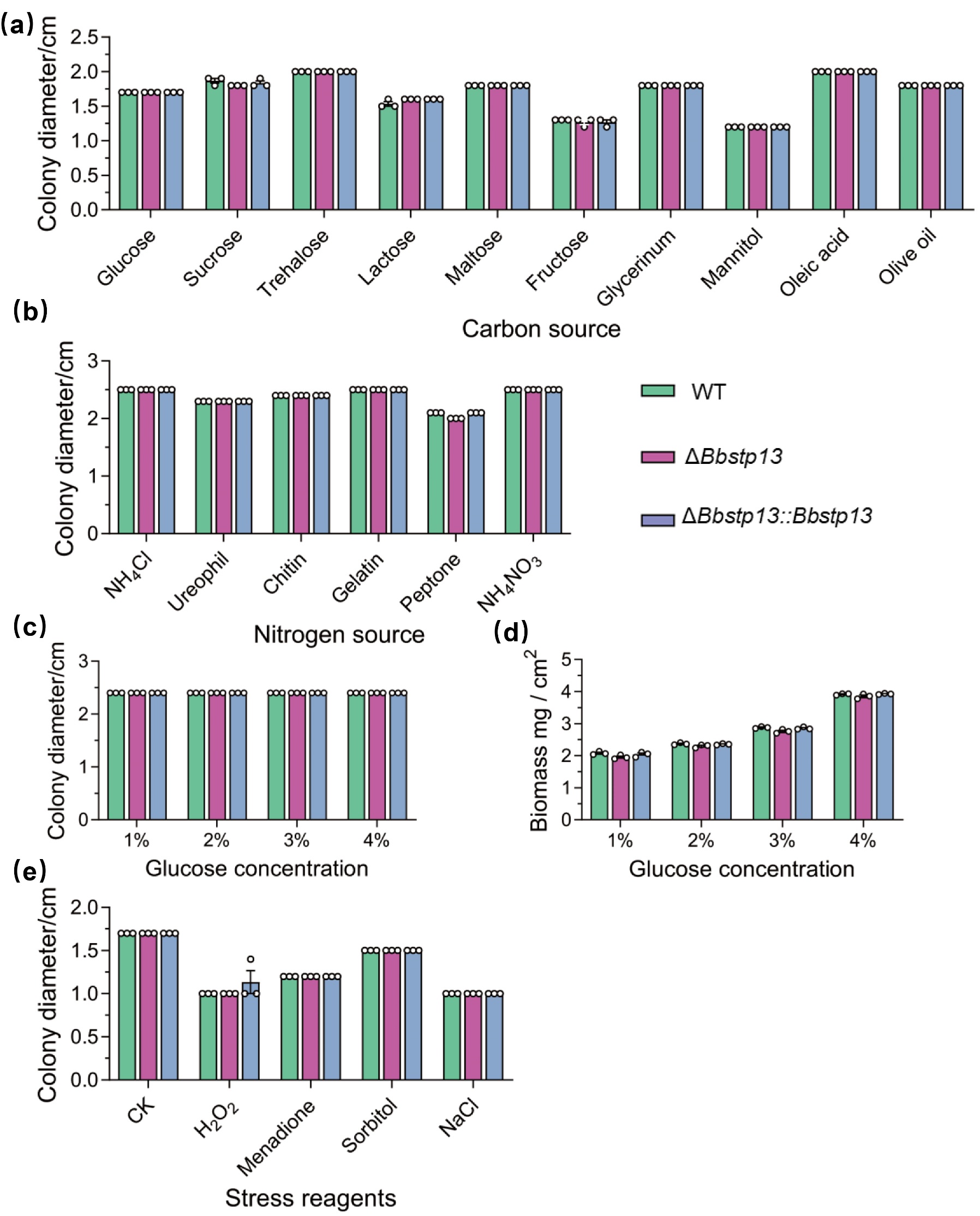


**Figure S4 Effects of BbStp13 on vegetative growth of *B. bassiana*.** (a) Vegetative growth on CzA medium with different carbon sources. The indicated carbon sources replaced the default nutrient, and conidia from each strain were inoculated onto the plates. (b) Vegetative growth on CzA medium with different nitrogen sources. The nitrogen sources were replaced with the indicated nutrients, and conidia were inoculated onto the plates. Colony diameters were measured after 7 days of incubation at 25°C. (c and d) Fungal vegetative growth and biomass under different glucose concentration. (e) Fungal vegetative growth under stress conditions. Mycelial growth was assessed under oxidative (2 mM H_2_O_2_, 2 mM menadione) and osmotic (0.5 M NaCl, 1 M sorbitol) stress on CzA plates. A conidial suspension (1 µL, 10^6^ spores/mL) was cultured at 25°C, and colony diameters were examined after 7 days of incubation.

**Table S1. Primers were used in this paper.**

| Primers | Paired sequences (5'-3')* | Purpose |
| --- | --- | --- |
| BbStp13GF/BbStp13GR | TCGCATTCAATCACAAACACCTTCAAAATGCCCATCGGCAATCTGTACGTC/CTCCTCGCCCTTGCTCACCATCTGCTTGTTGGGGGAAACCTCATGTTC | Cloning *BbSTP13* cDNA |
| RTBbStp13F/RTBbStp13R | CCGAAACCATTCCAAGAA/ AGAGGCAACAAATAGGTAC | qPCR for *BbSTP13* |
| 18SF/18SR | TGGTTTCTAGGACCGCCGTAA / CCTTGGCAAATGCTTTCGC | qPCR for 18s |
| BbStp13UF/BbStp13UR | GTACCGAGAAATCCCAAATCGTACCGAGAAATCCCAAATC/ AGCTCTCTTTGCAGGTGTTTAGCTCTCTTTGCAGGTGTTT | Amplifying 5′-fragment for BbStp13disruption vector |
| BbStp13DF/BbStp13DR | ATCTCACCTCCCTCCAAGCATCTCACCTCCCTCCAAGC/AGCACCAGAGCCCGAGTAAGCACCAGAGCCCGAGTA | Amplifying 3′-fragment for BbStp13 disruption vector |
| bStp13HF/BbStp13HR | ATCCGTCGACCTGCAGCCAAGCTTCGGAGCCGAAGACAGTTGCC/ ACACTAGTCAGATCTTCTAGTGTCAAAATAGCACCAGAGCCCGAGT | Cloning the BbStp13 full ORF for gene complementation |
| BbStp13JF/BbStp13JR | ACGGCGACAACTTCAATC / AAAGGGATGGTTCGGGTC | PCR detecting ∆*Bbstp13* |

* The underlined region is required for homologous recombination during plasmid construction.

**Table S2. Structural domain of sugar transporters in *B.bassiana*.**

|  | Domains | | | | | | | | | | | |
| --- | --- | --- | --- | --- | --- | --- | --- | --- | --- | --- | --- | --- |
| Proteins ID | Pfam:Glyco_hydro_88 | low_complexity_region | Pfam:Sugar_tr | Pfam:MFS_1 | Pfam:OATP | Pfam:TRI12 | transmembrane_domain | SCOP:d1an4a_ | LDLa | Pfam:PRKCSH-like | Pfam:PRKCSH_1 | Pfam:PRKCSH |
| XP_008599915 | 1 (121-429) | 2(465-476,592-603) | 1(618-1068) | 1(622-1017) |  |  |  |  |  |  |  |  |
| XP_008594993 |  |  | 1(61-529) | 1(65-443) |  |  |  |  |  |  |  |  |
| XP_008602488 |  |  | 1(64-518) | 1(61-468) |  |  |  |  |  |  |  |  |
| XP_008598992 |  |  | 1(6-439) | 1(3-363) |  |  |  |  |  |  |  |  |
| XP_008602796 |  |  | 1(22-502) | 1(23-455) |  |  |  |  |  |  |  |  |
| XP_008601910 |  | 4(72-90,162-174,184-198,731-749) | 1(223-664) | 1(227-664) |  |  |  |  |  |  |  |  |
| XP_008602047 |  | 1(555-566) | 1(26-498) | 1(21-330) |  |  |  |  |  |  |  |  |
| XP_008601379 |  |  | 1(19-479) | 1(17-403) |  |  |  |  |  |  |  |  |
| XP_008602107 |  |  | 1(17-473) | 1(17-426) |  |  |  |  |  |  |  |  |
| XP_008600631 |  |  | 1(50-503) | 1(53-428) |  |  |  |  |  |  |  |  |
| XP_008596081 |  |  | 1(18-428) | 1(29-324) | 1(64-213) |  |  |  |  |  |  |  |
| XP_008593716 |  |  | 1(17-476) | 2(21-263,282-499) |  |  |  |  |  |  |  |  |
| XP_008600414 |  | 1(521-533) | 1(9-490) | 1(6-370) |  |  |  |  |  |  |  |  |
| XP_008595795 |  |  | 1(11-472) | 1(15-313) |  |  |  |  |  |  |  |  |
| XP_008596332 |  |  | 1(42-519) | 2(46-376,330-537) |  |  |  |  |  |  |  |  |
| XP_008599131 |  |  | 1(40-539) | 2(42-375,337-562) |  |  |  |  |  |  |  |  |
| XP_008602655 |  |  | 1(27-505) | 1(25-457) |  |  |  |  |  |  |  |  |
| XP_008602800 |  |  | 1(30-512) | 1(35-454) |  |  |  |  |  |  |  |  |
| XP_008597302 |  | 1(2-14) | 1(33-502) | 1(37-451) |  |  |  |  |  |  |  |  |
| XP_008600873 |  |  | 1(25-493) | 1(26-370) |  |  |  |  |  |  |  |  |
| XP_008594910 |  |  | 1(24-496) | 1(28-448) |  |  |  |  |  |  |  |  |
| XP_008602712 |  |  | 1(1-402) | 1(1-339) |  |  |  |  |  |  |  |  |
| XP_008597352 |  | 2(3-27,32-43) | 1(97-550) | 1(92-501) |  |  |  |  |  |  |  |  |
| XP_008599745 |  |  | 1(57-513) | 1(61-460) |  |  |  |  |  |  |  |  |
| XP_008595615 |  | 1(40-49) | 1(65-525) | 1(6-282) |  |  |  |  |  |  |  |  |
| XP_008596251 |  | 1(7-13) | 1(27-482) | 1(31-430) |  |  |  |  |  |  |  |  |
| XP_008593994 |  |  | 1(46-507) | 1(43-348) |  |  |  |  |  |  |  |  |
| XP_008599655 |  |  | 1(44-498) | 1(37-448) |  |  |  |  |  |  |  |  |
| XP_008596363 |  |  | 1(21-479) | 1(18-338) |  |  |  |  |  |  |  |  |
| XP_008598649 |  | 1(533-554) | 1(23-484) | 1(27-322) |  |  |  |  |  |  |  |  |
| XP_008597258 |  |  | 1(39-522) | 1(37-394) |  |  |  |  |  |  |  |  |
| XP_008597817 |  |  | 1(1-174) |  |  |  |  |  |  |  |  |  |
| XP_008602173 |  |  | 1(77-259) | 1(83-486) |  |  |  |  |  |  |  |  |
| XP_008602667 |  |  | 1(56-247) | 1(65-471) |  |  | 1(521-543) | 1(480-519) |  |  |  |  |
| XP_008599055 |  |  | 1(24-444) | 1(13-396) |  |  |  |  |  |  |  |  |
| XP_008597966 |  |  | 1(18-459) | 1(22-376) |  |  |  |  |  |  |  |  |
| XP_008594554 |  |  | 1(42-418) | 1(46-469) |  |  |  |  |  |  |  |  |
| XP_008597264 |  |  |  | 2(658-773,770-1069) |  |  |  |  | 1(36-78) | 1(87-209) | 1(391-569) | 1(431-502) |
| XP_008596613 |  |  | 1(20-503) | 1(66-450) |  |  |  |  |  |  |  |  |
| XP_008598993 |  |  | 1(70-517) | 1(74-476) |  | 1(85-269) |  |  |  |  |  |  |
| XP_008603370 |  |  | 1(121-577) | 1(124-339) |  |  |  |  |  |  |  |  |
